# Lead editorial: Trials – using the opportunities of electronic publishing to improve the reporting of randomised trials

**DOI:** 10.1186/1745-6215-7-6

**Published:** 2006-03-23

**Authors:** Douglas G Altman, Curt D Furberg, Jeremy M Grimshaw, Peter M Rothwell

**Affiliations:** 1 Centre for Statistics in Medicine, Wolfson College Annexe, Linton Road, Oxford OX2 6UD, UK; 2 Department of Public Health Sciences, Wake Forest University School of Medicine, Medical Center Boulevard, Winston-Salem, NC 27157-1063, USA; 3 Clinical Epidemiology Programme, Ottawa Health Research Institute, and Department of Medicine, University of Ottawa, 1053 Carling Avenue, Ottawa ON K1Y 4EY, Canada; 4 University Department of Clinical Neurology, Radcliffe Infirmary, Woodstock Road, Oxford OX2 6HE, UK

## Abstract

This editorial introduces the new online, open access, peer-reviewed journal *Trials*. The journal considers manuscripts on any aspect of the design, performance, and findings of randomised controlled trials in any discipline related to health care, and also encourages the publication of protocols. Trialists will be able to provide the necessary detail for a true and complete scientific record. They will be able to communicate not only all outcome measures, as well as varying analyses and interpretations, but also in-depth descriptions of what they did and honest reflections about what they learnt.

*Trials *also encourages articles covering generic issues related to trials, for example focussing on the design, conduct, analysis, interpretation, or reporting.

## Introduction

'In 1963, Peter Medawar asked whether the scientific paper was a fraud. He argued that the research article was a "travesty... which editors themselves often insist upon" because it gives "a totally misleading narrative of the processes of thought that go into the making of scientific discoveries." A paper's fraud, Medawar insisted, lay mainly in its form. The importance of the form in which research is communicated, rather than its specific content, remains a neglected area of inquiry.' [1]

The creation of this new journal, *Trials*, has been stimulated by several parallel developments over recent years. First, there has been increasing recognition of the importance of randomised controlled trials (RCTs) as the most reliable source of evidence about the effects of health care interventions. Second, a growing realisation has developed in parallel that not all RCTs are equal, and that reliable results come most readily from those trials that have been conducted to the highest standards. Third, it is now widely accepted that those high standards cannot be assumed but have to be demonstrated by full and honest reporting of trials. It is not acceptable for readers to have to assume that certain procedures were adopted.

As interest in trials has increased, the importance of full reporting has become more widely recognised. Yet despite an increasing number of journals, paradoxically it seems to have become harder for authors to get adequate space in journals, especially traditional paper-based ones, to report fully the findings of their trials. Trial reports are thus unable to do justice to the huge contribution of trial staff and participants often over many years. Reviews of published trials consistently show that key information is frequently missing [2], although there is some evidence that adoption of the CONSORT Statement has led to improved reporting [3]. There is also growing evidence that such space pressures influence the way that researchers choose to write up their studies, with a bias in favour of selecting those outcomes that are statistically significant [4,5].

*Trials *has evolved from the journal *Current Controlled Trials in Cardiovascular Medicine*, and benefits from the hard work in establishing that journal. The scope has been broadened to cover all areas of health care and we will encourage and facilitate innovation in the style of publications. Continuity is maintained by the inclusion of one of the editors of the former journal (CF) in the steering group of the new journal. The main focus of *Trials *will be the publication of articles relating to the design, conduct, or results of particular randomised trials. We will in addition publish articles relevant to trials in general. We consider in turn aspects of these two main strands. The scope of the journal is summarised in Table 1.

**Table 1:** Scope of *Trials*

**Articles about a specific randomised trial**
Complete trial protocol
(An accompanying discussion of why the trial was designed the way it was is encouraged)
First report of trial findings
Expanded report of trial findings after a shorter version has appeared in another peer-reviewed, paper-based journal
Secondary analyses (e.g. health economic analyses, harms and other non-primary outcomes, sensitivity analyses)
Discussion of specific issues of the trial design
Description of particular aspects of the trial conduct, such as data collection, choice or measurement of outcomes, training of observers, data handling, organisational issues, etc
Lessons learned: "What we would do differently knowing what we know now"
Critical commentary on a trial report published elsewhere
**Articles about randomised trials in general**
Issues related to the design, conduct, analysis, interpretation, reporting or publication of randomised trials
Reviews of published articles describing randomised trials, relating to one or more of trial design, organisation, conduct, analysis, reporting, and interpretation
Issues relating to assessing outcomes, especially efforts to standardise outcomes in a particular medical field
**Commentaries**
Commentary to accompany articles published in Trials (usually commissioned)
Discussion of issues relating to randomised trials, especially topical matters

## Publications relating to specific randomised trials

*Trials *will consider manuscripts on any aspect of the design, performance, and findings of randomised controlled trials in any discipline related to health care, and will also encourage the publication of protocols. Trialists will be able to provide the necessary detail for a true and complete scientific record. They will be able to communicate not only all outcome measures, as well as varying analyses and interpretations, but also in-depth descriptions of what they did and honest reflections about what they learnt.

An especially important role for *Trials *is to make trial protocols available to everyone. A published protocol establishes intellectual property, allows discussion of methodological issues at greater length than is usually allowable, and can be referred to for further methodological detail when reporting the main trial results. Also, this public record of a trial reduces the risk of non-publication and may help to reduce the risk of inappropriate replication of trials. We will encourage authors to accompany their protocol with a discussion of why the trial was set up the way it was, indicating why alternatives were not adopted.

Authors will be expected to follow the recommendations of the CONSORT Statement [6]. *Trials *will give adequate space to present results in suitable detail. After evidence that trials reported as 'short reports' (500–600 words) had serious reporting deficiencies [7] the *Lancet *stopped using that format, but some journals continue. Indeed some journals specifically note that trials with 'negative' findings can be reported in brief, as if somehow those findings are less important. But even full-length reports of say 2000–4000 words frequently fail to include key information about methods and results. As Donald Mainland observed in 1938:

"Limitation of journal space and the expense of publishing numerous or elaborate tables usually prohibit full publication of data, except where these are few in number; and yet incompleteness of evidence is not merely a failure to satisfy a few highly critical readers. It not infrequently makes the data that are presented of little or no value." [8]

The results of randomised trials should be reported, regardless of what the findings were. We support the view expressed by Austin Bradford Hill almost 50 years ago:

"A negative result may be dull but often it is no less important than the positive; and in view of that importance it must, surely, be established by adequate publication of the evidence." [9]

A similar view was expressed by Iain Chalmers in 1990:

"Failure to publish an adequate account of a well-designed clinical trial is a form of scientific misconduct which can lead to those caring for patients to make inappropriate treatment decisions." [10]

And more recently the International Committee of Medical Journal Editors (2004) wrote:

"In return for the altruism and trust that make clinical research possible, the research enterprise has an obligation to conduct research ethically and to report it honestly." [11]

Compounding the problems of non-publication of some studies, especially those with non-significant results, it is clear that authors may report their results selectively, emphasising those that are statistically significant [5]. *Trials *will enable and encourage complete reporting of trial results, so that all the collected information can be made available. For this reason, *Trials *will consider for publication detailed, extended versions of reports of RCTs that have already been published in a conventional, short form.

Bearing in mind the dangers of over-summarising study data, we encourage graphical displays that show results for individual trial participants. We will also encourage authors to make available all (or some of) the raw data from the trials [12]. Such detailed reports, and especially the raw data, will be of great value for subsequent systematic reviewers, whose efforts are regularly devalued by the inadequate and even dishonest way in which trials are currently reported.

*Trials *will thus facilitate the publication of a series of linked publications from a single trial, beginning with the study protocol:

"Electronic publication of a protocol could be simply the first element in a sequence of 'threaded' electronic publications, which continues with reports of the resulting research (published in sufficient detail to meet some of the criticisms of less detailed reports published in print journals), followed by deposition of the complete data set. Not only would this approach allow alternative explorations of the data, it would help to address some of the growing concerns about research misconduct." [13]

A key element here, of course, is the availability of a unique trial identifier associated with trial registration [14]. Registration is a condition for publication in *Trials *of any article based on a specific trial.

*Trials *will be a forum for discussing other aspects of specific randomised trials, as an important element in the full publication of a trial or as an educational resource, or both. Such information may provide insight into scientific or practical problems (e.g. recruitment, choice of outcome measures, resolution of logistic challenges, data monitoring) and thus may have value for those planning and conducting future trials.

In addition, we believe that there is scope for new and better ways to report the findings of trials. *Trials *will develop and refine innovative approaches to improving communication about trials, in particular to make the article's message comprehensible to a variety of reader groups. Here we anticipate taking advantage of the ingenuity of authors and the fine advisory team that we have assembled.

*Trials *will have a special section that will present commentaries on, and critiques of, trial reports published in other journals, with neither the time nor word constraints of letters in those journals. We will invite the authors of the original article to respond, and will indicate if they choose not to do so.

## Publications relating to randomised trials in general

Articles relating to trials in general are of particular value as they may have a considerable beneficial impact on many future trials.

*Trials *encourages articles covering generic issues related to trials, for example focussing on the design, conduct, analysis, interpretation, or reporting of RCTs. We especially encourage discussions of issues relating to assessing outcomes, including efforts to standardise outcomes in a particular medical field. We will also consider articles relating to broader issues, such as data monitoring, trial registration, and the ethical or philosophical underpinnings of trials.

*Trials *welcomes reviews of published articles describing RCTs relating to one or more of the following: trial design, organisation, conduct, analysis, reporting, and interpretation. We will also publish the results of empirical methodological studies relating to trial methods and discussions of generic methodological issues.

## Comments

The medical literature is vast and it is impossible to keep up with the deluge of new research articles. So is the relentless increase in the number of journals a good thing? We believe that there is a clear need for *Trials*. It fills a particular function that we do not believe exists at present. As it is open access there will be no restriction on the availability of the information. With free online databases, identification of and access to articles is no harder with many journals than with just one, as long as those journals are freely available.

Because *Trials *is online-only it is not subject to the space constraints of paper journals. This will enable us to move away from the traditional format of journal articles. We know that readers vary in their needs, from those who want a rather short summary of the study to those who wish to see absolutely everything. But even the former group will wish to be reassured that the full information is available (and indeed has been assessed as part of peer review). Over time we aim to help to satisfy such variation.

Finally, *Trials *will provide a venue for publishing full trial details to address the concerns expressed by Richard Feynman in his Nobel Lecture in 1966:

"We have a habit in writing articles published in scientific journals to make the work as finished as possible, to cover up all the tracks, to not worry about the blind alleys or describe how you had the wrong idea first, and so on. So there isn't any place to publish, in a dignified manner, what you actually did in order to get to do the work." [15]

We invite authors who share our perspective to do justice to their trials and submit to *Trials*.
